# Treatment of Uterine Fibroids*Read before the Hennipen County Medical Society, February 6, 1899.

**Published:** 1899-03

**Authors:** F. A. Dunsmoor

**Affiliations:** Minneapolis


### TREATMENT OF UTERINE FIBROIDS.*

BY F. A. DUNSMOOR, M. D., MINNEAPOLIS.

Hardly a day passes that I am not consulted by a patient who has a uterine fibroid of greater or less size. Some writers state that one woman in every ten has a uterine fibroid. Others make the percentage higher, even fifteen per cent. The remarkable frequency of this disease and the probability of amelioration of many of its most distressing symptoms by simple methods of treatment, induced me to select the topic of "Treatment of Uterine Fibroids" for this brief paper.

With all the opportunities offered to the medical investigator and the pathologist, the specific cause for fibroid has not been discovered, although it must be remembered at the present day that heredity is an important factor. The growths appear so fequently in sterile and unmarried women as to eliminate pregnancy as a cause, although the tumor is often developed with the foetus in utero. The decade between twenty-eight and thirty-eight years is the one in which the greatest number of fibroids is found. Dr. Emmett believes that the unmarried woman at this period is "twice as liable to fibr[illegible]ds as the married woman, sterile or fruitful."

He says, "It seems as if it were the purpose of nature that the uterus should undergo a change, dependent upon pregnancy or lactation, about every three years throughout the child-bearing period, and that if the uterus is not physiologically occupied in child-bearing, the fibroid will be more rapidly developed."

Brooks Wells says, "myomata of the uterus are more common in old maids than in married women." It is an undeniable fact that negresses are more liable to fibroids than white women. A variety of symptoms arise from growths which vary in size from that of the smallest shot to the extreme capacity of the abdominal coverings, but it is beyond the province of the writer to consider, at this time, symptoms, complications and diagnosis other than these conditions bear upon the treatment, but once the diagnosis is made, we believe it to be the duty of the physician to begin that form of treatment which seems the most applicable to the type of fibroid present, even if the tumor is creating no disturbance at the time. It is admitted that fibroids are benign growths, and the question of the fibroid degenerating into sarcomatous or carcenomatous conditions is generally disbelieved.

We will consider medical, electrical and surgical treatment, but under these separate headings we are obliged to resort to special procedures for the different types of fibroids.

### SUBMUCOUS.

Submucous fibroids are a type, which in my experience, are found either singly or associated with some other type, in

*Read before the Hennipen County Medical Society, February 6, 1899.

twenty-five per cent. of all the cases. In this variety the patient usually applies for a relief from a dragging sensation, persistent leucorrhoea or alarming hemorrhage, and all the symptoms may be present and due to a growth so small in size as to scarcely enlarge the uterus, and the tumor itself be undiscovered save by intrauterine examination. It will be necessary to use vaginal disinfectants of which lysol is the best example, when examination of the uterine cavity is to be made. The installation, semi-weekly, within the uterine canal, of a drop of Churchill's tincture of iodine, turpentine or a weak solution of chloride zinc or, if either is not easily obtainable, an application of the tincture of the muriate of iron is especially beneficial. Internally, I should give uterine sedatives like bromide of sodium, black haw, belladonna or hyoscyamus, and if the uterus is soft and flabby, uterine and muscle tonics like ergot, strychnia and hydrastis. Tampons which are saturated with glycerine holding in suspension alum, zinc, turpentine or hydrastis should be applied around the cervix.

Associated with these remedies, when the growth is large enough to be discovered upon examination, with or without dilatation of the cervical canal, the tumor should be removed by curettage, serrated spoon, snare or forceps. Following such manipulation the uterine cavity should be swabbed out with iodine and carbolic acid ten per cent., or any of the astringents named in the first class.

During an attack of flooding the administration of coal tar derivatives, particularly antipyrine, or the injection of a saturated solution within the uterine cavity has a decidedly beneficial effect. As in any other disease where the patient has lost much blood, it will be necessary to resort to general tonics, and particularly, those which supply the sanguinary fluid. Iron, manganese and hypophosphites, associated with arsenic and muriate of ammonia, are among our choice of remedies.

If constipation is present it should be controlled by interrupted doses of mild chloride of mercury with cascara sagrada. We have ofttimes seen hypodermic injection of one-hundredth of a grain of atropine have a decided effect in quieting the bearing-down sensation and diminishing the hemorrhage. If the patient is particularly wakeful and nervous give one-half a drachm of chloral per rectum, and if the patient is not asleep in three hours the dose is repeated.

### INTERSTITIAL.

In the interstitial type, in which the enlargement is usually symmetrical with the shape of the body of the uterus, the patient frequently complains of symptoms associated with the submucous variety, but more likely is disturbed on account of pressure against the abdominal viscera, associated with some cystitis, bowel disturbance, backache and the bearing-down sensation which so frequently accompanies the menstrual flow. Before the tumor is sufficiently large to have escaped from the pelvic cavity, the patient complains of reflex pain in the occiput or in the temporal region.

Medical treatment of this class of cases combines usually the administration of bromide of sodium with the exhibition of the thyroid extract, three or four times a day, in doses increasing from five grains. In my own experience this is the only class in which ergot has a decidedly beneficial effect. It must be given continuously and, when associated with the thyroid extract and the application of the positive electrode in the uterine canal and the large clay cathode or the Martin membranous abdominal electrode directly over the abdomen, secures the greatest number of cures over any method, aside from the surgical procedure. The current which I employ is taken from the dynamo in the office in the New York Life building, voltage, one hundred and ten, and the current, usually begun at twenty-five and run up as high, sometimes, as two hundred milliamperes.

### INTRAMURAL AND SUBPERITONEAL.

Intramural and subperitoneal fibroids are affected very little by medical remedies. The greatest benefit to be hoped for is secured by the administration of the thyroid extract and electricity, my experience in this variety being exactly opposite to that of Prof. Franklin H. Martin in the Post Graduate School of Chicago. When I first began the use of electricity in the treatment of fibroids, the positive pole terminated in a needle which was introduced into the growth through Douglas' cul-de-sac whenever possible, and the abdominal electrode used as above.

Later, I used the vaginal electrode as furnished by the Macintosh Company of Chicago, and am obliged to confess that I was disappointed in the results from either, or the combined methods.

Exactly why the use of electricity should be beneficial in the treatment of fibroids is not explainable by the writer, but it is unnecessary for me to read to this intelligent body the vast number of names of prominent men of our profession, of undoubted veracity, who report astonishing cures by this method. While I can not say that I have cured, in the sense of having caused the tumor to completely disappear by this method, many fibroids have ceased their rapid growths, and patients who were having alarming hemorrhages have been much benefited by this treatment in my office.

It is well known that the action of the anode in the animal tissue is to dry up and drive the blood from the surface to which it is applied, while the reverse is true of the negative pole. Hence we understand this much of the beneficial effect of galvanism to the uterine cavity in hemorrhagic cases. The positive pole is also decidedly antiseptic and lessens the caliber of the blood-vessels in its proximity.

Dr. Martin believes that the acid generated by the positive pole combines with the copper of the electrode to form salts, which, by cathoresis, are driven into the uterine tissue, thus producing a true styptic effect. There are many women who are frightened to distraction at the mere suggestion of a hysterectomy, or indeed, of any surgical procedure, but eagerly submit, with the greatest amount of fortitude, to the application of the powerful electric current up to the point of pain, having semi-weekly sessions lasting from ten to fifteen minutes at a time without a murmur. In these cases where the discharge is purulent or there is a profuse catarrh, the canal is first curetted and iodine, zinc or muriatic acid applied.

I have often reduced the size of goitres by the electric method. Illustrating the fact that the beneficial effect of this current is not limited to uterine fibroids, I will mention the case of a patient from whom I removed a large ovarian tumor, who was also afflicted with excessive enlargement of the lymphatic glands of the neck, and on my suggestion to have them treated, promptly told me that as soon as she was able to return home, she, herself, could control them by the application of a galvanic battery which she possessed.

### SURGICAL TREATMENT.

From the laity and the general practitioner, and also from a large majority of the surgeons, comes the demand for conservatism in the treatment of all abdominal growths, and the demand is certainly applicable to uterine fibroids, so that now, often operations of much less magnitude than a complete hysterectomy are made for the relief or cure of these tumors. Mr. Tait believes that in certain selected cases the removal of the uterine appendages is sufficient to cure the tumor and its symptoms. Tait, Hegar and Battey conceived the idea of an operation for the removal of the appendages for the purpose of creating an artificial menopause, and each published his idea in the year 1872.

While it is known that many cases of fibroids have increased in size or undergone distinct degeneration at the time of the change of life, still, in the majority of cases, fibroids have improved without treatment, at, or after this period, and while the operation for the removal of the tubes or appendages frequently fails to terminate menstrual life, still, the operation has often proved sufficient, in good hands, to satisfactorily control the growth of fibroids in the uterus. It must be admitted that it has also been as complete a failure for this purpose, as it has for the establishment of the change of life.

In our own country the habit is growing, to make, in all pedunculated, subserous and many intramural cases, what is known as myomectomy, or to treat the tumor exactly as if it were a growth on the outside of the body, instead of connected with the uterine organs. Dr. Martin has also devised an efficient operation for the purpose of shutting off the blood supply of the uterus and its fibroids, which has proved very successful in his hands, and which is to be commended to the profession. I quote from Dr. Martin's book a description of the operation as follows: "The ligation of more or less of the broad ligament of the uterus with its vessels and nerves, the extent of the ligation depending upon the result sought, from a simple ligation of the base of the ligament, including the uterine arteries and branches of both sides, without opening the peritoneum, to a complete ligation of the ligament of one side, including both uterine and ovarian arteries, with partial ligation of the opposite ligament without opening the peritoneal cavity, if possible, but by doing so if necessary.

"The results sought in the operation are, first, to check uterine hemorrhages by cutting off blood-channels, and secondly, to produce atrophy of the fibroid by first depriving it of nourishment through the blood vessels, and thirdly, changing the nutrition of the uterus by interfering with its nerve supply."

After the established antiseptic precautions the technique of the operation is as follows: "The uterus is drawn down in order to put the broad ligaments on the stretch, and then drawn to the right side so as to expose the left vaginal vault. The mucous membrane of the vagina at the utero-vaginal fold on the left is then caught with a tenaculum and incised with a pair of curved scissors. One blade is allowed to enter beneath the mucous membrane and a curved incision one and one-half to two inches long, is made over the broad ligament and at right angles to it. By means of the index fingers of both hands the operator now separates the vaginal tissue of the broad ligament and carefully separates the broad ligament in front from the bladder for a height of two inches, and laterally for nearly the same distance. The bladder should be carefully separated in this way in order to avoid the danger of wounding the organ, and by pushing the separation laterally the ureter is forced out of danger. One then carefully separates the broad ligament posteriorly to the same height as in the front, without, if possible, penetrating the peritoneum. Now, by passing one finger behind the other in front, the whole base of the broad ligament, representing two-thirds of its bulk, can be grasped for a distance of an inch to an inch and a half from the uterus. In this grasp one can easily feel the throb of the main trunk of the uterine artery, and occasionally several branches. The curved pedicle needle is then passed, armed with No. 10 silk, strong pyoktanized catgut or kangaroo tendon, and guided by the index finger of the left hand, is made to penetrate through the broad ligament. The ligature is drawn through, the needle removed and the base of the broad ligament is thoroughly ligated at a distance of an inch or more from the uterus. The ligature is cut short, leaving it buried in the tissues. The broad ligament is treated in the same manner."

Dr. Martin intends this operation for controlling the interstitial fibroid of moderate size, and particularly, those fibroids appearing late in menstrual life and in such cases where the major operation is particularly undesirable. It is certainly the method to be selected for the class of cases which has been practically exsanguinated by profuse hemorrhage until the patient is so exhausted that one does not dare to perform an abdominal hysterectomy.

After all that has been said or done that is possible, for the relief of the patient with fibroid by simpler or milder means, this one great fact remains that the one absolute, sure cure for uterine fibroids is hysterectomy. This operation formerly was considered so dangerous that the most intrepid surgeon pushed his work in this direction with the gravest apprehensions. Now, with the advancement of surgical knowledge, antiseptics, technique and experience, it has become almost, if not quite, as safe an operation as a double ovariotomy with which it is not unfrequently associated.

All those cases which seriously interfere with the mind or bodily comfort, either by pressure effects or hemorrhage, or interfere with domestic happiness and are not relieved by any of the methods described, should certainly be cured by this operation. Once this is decided upon we still have the question of the route and the method to be employed. In women who have borne children, and if the tumor does not extend beyond the pelvic cavity, the vaginal route is to be employed, although it is the writer's belief that those tumors which are so large as to require morcellement before they can be removed per vaginam, should be extirpated by the abdominal route, since, in the hands of a large majority of surgeons of undoubted experience, the latter will be shorter and safer.

Having decided to remove per vaginam, the writer believes that instead of following the practice of making strong traction upon the cervix through the vagina, enucleating the mass as fast as it can be extricated from the vulva, that exactly the contrary method should be applied. First, the cervix is completely girdled by an incision extending through the vagina, passing close to the uterine neck. The incision is extended if necessary, three-fourths of an inch on either side, then the tumor and the cervix are pushed as deeply into the pelvis as possible, while the fingers separate the bladder by pushing it from the tumor or uterus itself, until the peritoneum is reached. The same procedure is adopted through the posterior cul de sac, then the uterine arteries and the lower portion of the broad ligament are tied and divided close to the uterus. If there is difficulty in removing the growth through an incision as described, the opening may be enlarged by incising the vagina in the median line antero-posteriorly. If this opening should still prove insufficient, the uterus may be divided through the center and one-half delivered at a time.

In about one case in ten after vaginal hysterectomies, I unite the divided surface at the extremity of the vaginal vault by suture. Ordinarily after the pelvis has been cleansed, the edges of the wound are simply approximated and the vagina loosely filled with iodoform gauze and the patient put to bed, experience having shown that the wound unites perfectly and without complications. Tumors which are sufficiently large to have a diameter greater than that of the pelvis, are invariably removed by the abdominal route, by the writer. The length of the incision depends upon the size of the tumor, and in extreme cases, may extend from the pubes to the ensiform appendix, and in one enormous growth, upon which I operated, lateral incision was required in order that the huge tumor could be extricated.

In earlier operations, as soon as the uterus and tumor were freed from their bed and pulled up through the abdominal wall, the serre nœud was thrown around the mass as close as possible to the bladder, and when this garrote was sufficiently tightened, two long steel needles or hat-pins were driven through the cervix, above the constricting wire, while the amputation was made above the transfixing pins, and the peritoneum immediately below the wire was stitched to the mural peritoneum and the wound closed. There is no doubt that in many growths this is the safer and more rapid procedure. However, in the last fifty cases done by the writer, the method has been that of Baer or Kelly, in which the amputation is made after the ligation of the broad ligaments and the vessels between them, the stump covered in by the peritoneum after the antero-posterior walls have finally united by buried catgut sutures.

It is not my intention to consider, to-night, the many varieties of hysterectomies which may be made, since the first intention was simply to call attention to various successful methods of treating the fibroids before coming to the "dernier resort."—*Northwestern Lancet.*

